# Automated analysis of speech as a marker of sub-clinical psychotic experiences

**DOI:** 10.3389/fpsyt.2023.1265880

**Published:** 2024-02-01

**Authors:** Julianna Olah, Thomas Spencer, Nicholas Cummins, Kelly Diederen

**Affiliations:** ^1^Department of Psychosis Studies, Institute of Psychiatry, Psychology and Neuroscience, King’s College London, London, United Kingdom; ^2^Department of Biostatistics & Health Informatics, Institute of Psychiatry, Psychology and Neuroscience, King’s College London, London, United Kingdom

**Keywords:** psychosis, NLP, paralinguistics, speech, sub-clinical psychosis, machine learning

## Abstract

Automated speech analysis techniques, when combined with artificial intelligence and machine learning, show potential in capturing and predicting a wide range of psychosis symptoms, garnering attention from researchers. These techniques hold promise in predicting the transition to clinical psychosis from at-risk states, as well as relapse or treatment response in individuals with clinical-level psychosis. However, challenges in scientific validation hinder the translation of these techniques into practical applications. Although sub-clinical research could aid to tackle most of these challenges, there have been only few studies conducted in speech and psychosis research in non-clinical populations. This work aims to facilitate this work by summarizing automated speech analytical concepts and the intersection of this field with psychosis research. We review psychosis continuum and sub-clinical psychotic experiences, and the benefits of researching them. Then, we discuss the connection between speech and psychotic symptoms. Thirdly, we overview current and state-of-the art approaches to the automated analysis of speech both in terms of language use (text-based analysis) and vocal features (audio-based analysis). Then, we review techniques applied in subclinical population and findings in these samples. Finally, we discuss research challenges in the field, recommend future research endeavors and outline how research in subclinical populations can tackle the listed challenges.

## 1 Introduction

Psychotic disorders, such as schizophrenia and bipolar disorder, represent a significant challenge in mental health research and clinical practice. Identifying individuals who are at risk of developing these disorders or who may exhibit subclinical psychotic experiences is crucial for early intervention and preventive strategies. Traditional approaches to assessing psychotic symptoms have relied on subjective clinical interviews and self-report questionnaires, which are inherently limited by their reliance on patient insight and recall accuracy. However, recent advancements in technology and computational linguistics have paved the way for novel and more objective methods of evaluating mental health, specifically through the analysis of speech. Given the strong connections between speech and psychosis, abnormalities have important implications for diagnosis, assessment, prevention, and treatment – to the extent that in one of the pioneering reviews of the field, the authors stated that “speech may offer one of the most informative collections of features for predicting psychosis” (1).

Here, we aim to explore the emerging field of automated analysis of speech as a potential marker of subclinical psychotic experiences. By leveraging machine learning algorithms, paralinguistic analysis and natural language processing techniques, researchers have begun to uncover subtle linguistic patterns and acoustic features that may be indicative of underlying psychotic symptoms. This innovative approach holds promise for enhancing our understanding of psychosis risk, early detection, and treatment outcomes. Automated speech analysis, however, has been rarely applied in sub-clinical populations – eventhough it could help researchers overcome limited sample sizes, widen the scope of research, enable the longitudinal observation of the emergence of speech changes in psychotic disorders and explore potential risk and protective factors. This review aims to facilitate such work and serve as an introductory guide to speech analysis in sub-clinical research. We will review, explain and summarize relevant concepts, techniques and research approaches, and identify current opportunities and challenges to inform future work.

## 2 Psychosis and sub-clinical psychotic experiences

**Figure 1:**
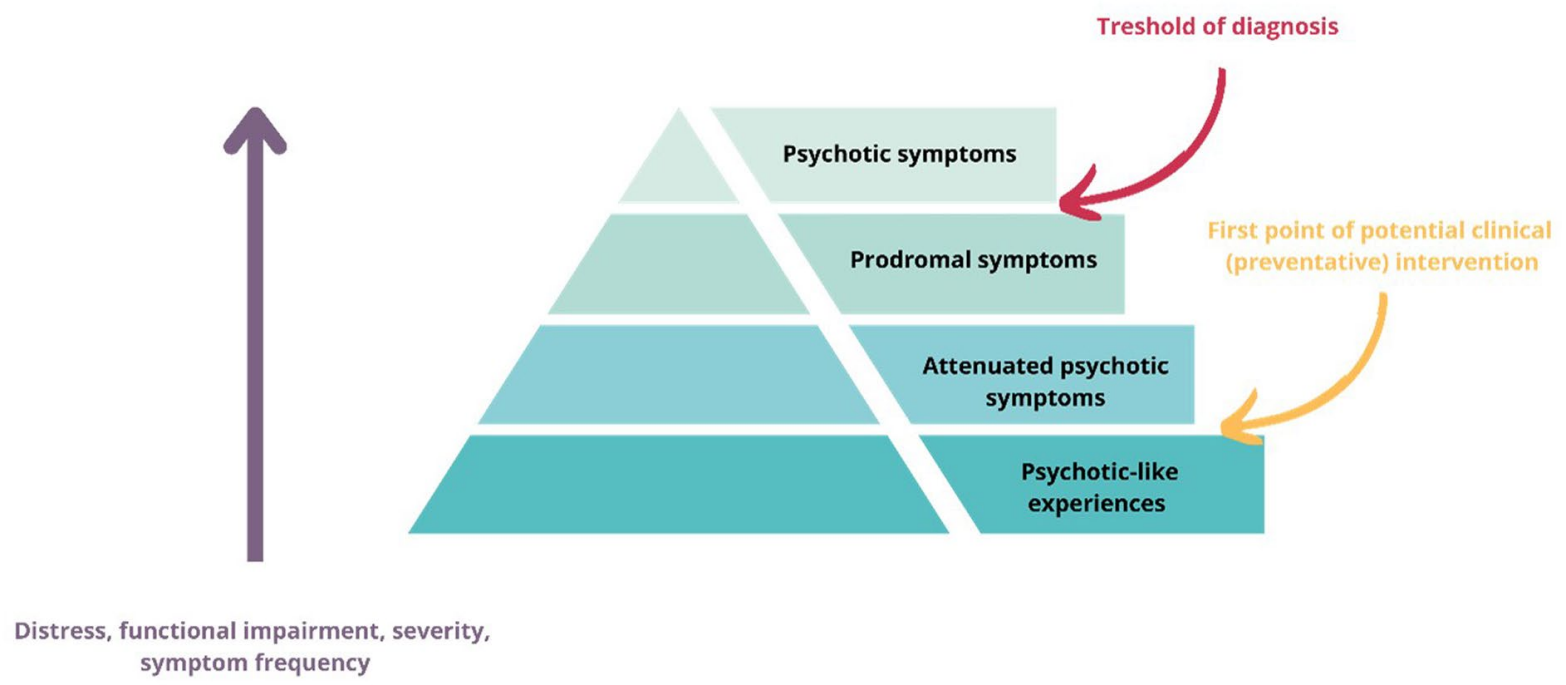
Illustration of the psychosis continuum.

Psychosis, a debilitating mental health condition characterized by a loss of contact with reality, has long been a subject of extensive research and clinical interest (2). However, it is increasingly recognized that the continuum of psychotic experiences extends beyond the clinical threshold, encompassing a broader spectrum of subclinical symptoms alongside with subtle alterations in neurodevelopment, perception, cognition, and affect. The psychosis continuum concept describes psychotic symptoms occur on a spectrum, with varying degrees of severity and impairment, as well as distress and help/seeking behavior (3) and emphasizes that psychotic symptoms can be present in non-clinical populations and that there is a gradual transition from subclinical symptoms to clinically significant psychosis in some individuals (2) (Figure 1). Subclinical psychotic experiences refer to milder forms of psychosis-like phenomena and can manifest as perceptual abnormalities, delusional ideation, or disorganized thinking, albeit at a lesser intensity or duration compared with clinical-level psychotic symptoms (4). These experiences are often transient, infrequent compared to those seen in people with diagnosed psychotic disorders, but they can still impact psychological well-being, quality of life and functioning (4, 5). Although authors provide varied and partly overlapping definitions, subclinical symptoms usually cover psychotic-like experiences, attenuated psychotic symptoms, prodromal symptoms and being at clinical risk of/ at ultra-high risk of psychosis. In this review, we apply that as a definition of subclinical symptoms/experiences.

Importantly, subclinical psychotic experiences are associated with an increased risk for transitioning to clinically significant psychosis (6)- therefore the examination of subclinical psychotic experiences could aid in identifying individuals at increased risk of developing a full-blown psychotic disorder (3, 6–8).

Studying psychotic-like experiences in the general population, as opposed to solely focusing on clinical samples, holds several important benefits for research and clinical interventions that we detail in Table 1.

**Table 1:** Benefits of studying subclinical psychotic symptoms and psychosis-like experiences.

Benefits of studying subclinical psychotic symptoms and psychosis-like experiences
**Understanding the spectrum of experiences**	By studying the general population, researchers can gain insights into a broader range of experiences, from mild and transient to those that might be indicative of underlying mental health conditions.
**Identifying risk factors and protective factors**	Examining psychotic-like experiences in the general population could allow us to identify risk factors that may contribute to the development of more severe psychotic disorders. Additionally, studying individuals who experience these phenomena but do not progress to clinical psychosis can offer insights into protective factors that might prevent the onset of full-blown psychotic disorders.
**Epidemiological data**	General population studies can provide valuable epidemiological data, such as the prevalence, incidence, and distribution of psychotic-like experience which is crucial in understanding the overall impact of these experiences on society and for public health planning.
**Uncovering subclinical patterns**	Psychotic-like experiences in the general population may manifest in subtle ways that individuals may not even recognize as relevant to mental health. By investigating these subclinical patterns, researchers can gain a deeper understanding of early-stage symptoms, potentially leading to earlier detection and intervention.
**Reducing selection biases**	Clinical samples may not fully represent the entire spectrum of individuals experiencing psychotic-like phenomena. Focusing solely on those seeking treatment may introduce selection biases and limit the generalizability of findings. Studying the general population helps to reduce such biases and provides a more representative sample.
**Making observations without the confounding effect of antipsychotic medication**	Clinical samples typically consist of individuals receiving treatment for psychosis, often including antipsychotic medication. While these medications can effectively manage symptoms, they can also modify the expression and intensity of them. Observations in general population can provide insights into the unaltered progression, variability, and potential outcomes of psychotic-like experiences, enhancing the validity and generalizability of research findings.
**Examining associations with other factors**	General population studies can explore associations between psychotic-like experiences and various demographic, social, and environmental factors via heterogenous and large samples. This can shed light on potential triggers or contributors to these experiences, providing a more holistic understanding of their etiology then studying clinical research alone.
**Development of preventive strategies and public health planning**	Insights from general population studies can aid in the development of targeted preventive strategies to reduce the risk of individuals progressing to clinical psychotic disorders.

## 3 Language and psychosis

### 3.1 Speech alterations in psychosis

Speech abnormalities are a prominent feature of psychosis, encompassing both positive and negative symptoms. Positive symptoms are an addition, excess or distortion of normal function (in formal thought disorder these could be neologisms, tangential thoughts, derailment, incoherence) and negative symptoms where there is a reduction or absence of normal behaviors (for formal thought disorder these could be, e.g., poverty of speech, reduced variation in prosody) (9). Specifically, semantic, and structural measures of speech coherence are strongly associated with formal thought disorder and are already present prior to illness onset (9–11). Negative symptoms, on the other hand, manifest as poverty of speech, alogia, and reduced verbal fluency (12–14). These symptoms can manifest in vocal features like a slower rate of speech with shorter utterances, more pauses, reduced variation in frequency or in language use, like in decreased density of ideas (15). In addition to these speech alterations, individuals with psychosis may exhibit other speech-related phenomena such as perseveration (continual involuntary repetition of a thought), neologisms (the coining or use of new words), clang associations (groupings of words that are based on similar-sounding sounds, even though the words themselves do not have any logical reason to be grouped together), and echolalia (the unsolicited repetition of utterances made by others) (12, 16) (Table 2).

**Table 2:** Connection between speech and psychosis.

**Connections between speech and psychosis**	
**Speech disturbances in psychosis**	- Positive symptoms: positive thought disorder, clang associations, neologisms. - Negative symptoms: Poverty of speech, alogia, and reduced verbal fluency. - Other speech-related symptoms: Perseveration, echolalia.
**Language and semantic processing**	- Impairments in semantic processing and word comprehension. - Disruptions in discourse coherence and cohesion. - Difficulties in producing coherent narratives.
**Prosody and paralinguistic features**	- Abnormalities in prosody, including monotone speech, reduced pitch variation, and inappropriate intonation. - Impaired emotional expression and affective prosody. - Nonverbal communication deficits, including reduced gesturing and facial expressiveness.
**Neurobiological correlates**	- Degree centrality from resting state functional imaging and cortical gyrification index correlate with speech connectivity. - Coherence is associated with superior temporal activation of the brain in psychotic patients. - Mean length of utterance is associated with integrity of white matter in language tracts in psychotic patients. - Speech dysconnectivity is hypothesized to emerge from cerebral dysconnectivity in psychosis.

### 3.2 Language and semantic processing deficits

Psychosis is frequently associated with impairments in semantic processing and word comprehension. Studies have shown deficits in semantic processing tasks, including reduced semantic priming effects and impaired word recognition (17). Furthermore, disruptions in discourse coherence and cohesion have been observed, leading to difficulties in maintaining coherent and cohesive conversations (18). Individuals with psychosis may also face challenges in producing coherent narratives, exhibiting fragmented and disorganized speech (18, 19) (Table 2).

### 3.3 Prosody and paralinguistic features

Prosodic abnormalities are common in individuals with psychosis and include monotone speech, reduced pitch variation, and inappropriate intonation (20–24). Impaired emotional expression and affective prosody, such as difficulty conveying appropriate emotional tones, have been observed. These alterations go beyond language as deficits in nonverbal communication, characterized by reduced gesturing and facial expressiveness are also present (25–27) (Table 2).

### 3.4 Neurobiological measures

Neuroimaging studies have identified neural correlates of speech disturbances observed in psychosis, implicating altered activation and integration (i.e., connectivity) of language-related brain regions (28). Although further research is needed to map the complexity of neural correlates of speech alterations, first findings in the field suggest that speech connectedness correlates with alterations in functional as well as developmentally relevant structural brain markers of psychosis (degree centrality from resting state functional imaging and cortical gyrification index) (29). In psychotic disorders, automated marker of coherence is associated with superior temporal activation, and mean length of utterance is associated with integrity of white matter in language tracts (28, 29) (Table 2).

### 3.5 Clinical application and considerations

There is evidence to suggest that speech features could serve as valuable markers in the diagnostic process, aiding in the identification and differentiation of psychotic disorders. For example, automated analysis of semantic and acoustic abnormalities can distinguish individuals with schizophrenia from healthy controls with an accuracy ranging from 70 to 99%. Also, differences speech connectivity able to discriminate between bipolar disorder and schizophrenia (30).

Furthermore, speech-based markers have shown promise in predicting clinical outcomes and treatment response, potentially enabling the development of more personalized and targeted interventions. For example, researcher could classify the diagnosis of psychotic disorders and severe negative symptoms 6- months in advance or predict who will develop psychotic disorder from ultra-high-risk state with 85% accuracy using automated analysis of speech (30–32). Also, in a longitudinal cohort of children with an increased genetic predisposition for psychosis researcher could predict who will develop schizophrenia 10 years later with 90% accuracy based on the manual analysis of the interview transcripts (33). Regarding treatment response, preliminary results of an ongoing study suggest that automated analysis of acoustic changes can predict relapse 1 month in advance with high accuracy (34).

For any form of application, we need to analyze speech in a quick, replicable, systematic and complex way that is ideally automated and scalable. Fortunately, advances in technology, including Natural Language Processing, acoustic analysis, signal processing, automated speech recognition and machine learning make speech a suitable signal for large-scale clinical application. In the next section, we review these techniques.

## 4 State-of-the art approaches to the automated analysis of speech in psychosis research

Research efforts that use automated analysis and assessment of speech in psychosis can be grouped into five categories based on their technical approach:

1. Semantic coherence and semantic density based on Latent Semantic Analysis or word-embedding.
2. Syntactic complexity and syntactic changes based on Part Of Speech Tagging.
3. Speech connectivity based on graph theory applied on text/spoken language.
4. Acoustic features, grouped into temporal, spectral, loudness, frequency features. They are often extracted using signal processing softwares like OpenSmile (free for research use) or Praat (open source). Applied features are often coming from predefined feature sets, designed to capture emotional information.
5. Deep Neural Networks – applied to audio data or spectrograms.

In the first four approaches, researchers first extract predefined features that have been associated with psychosis symptomology and speech deficits and apply statistical tests, machine learning algorithms like ensemble learning and shallow learning on these features – which makes the findings more explainable and the models easy, resource-efficient and quick to train. Approaches applying Deep Neural Networks (DNN-s) on the other hand do not extract predefined features from the data but allow the models to learn the abstract mathematical representation of informative patterns in speech without human knowledge in the loop. This approach gained significant success in AI research and application, generally overperforming the previous methods if sufficient training data were provided. For example, in psychiatric context, de Boer et al. used ensemble learning to discriminate between schizophrenia patients and healthy control groups based on vocal parameters of speech and reached 86% accuracy in the task (35). Fu et al. (36) used a DNN architecture on the same classification problem and reached 98% accuracy (36). Studies that aimed to detect major depression from speech reached 0.46–0.96 F1 score (a performance metric ranging from 0 to 1, where values closer to 1 indicate better performance) when applied shallow learning or ensemble learning techniques while studies that applied DNN reported 0.6–0.99 F1 scores (37). The reason behind this is the general scalability of the model in terms of digesting and integrating new information in a nuanced way and the ability to learn patterns that researchers might had not have considered. On the other hand, this approach reduces explainability of the models, requires significantly more training data, resources and time and arguably provides less clinical insight (38, 39). Another important limitation of DNNs is their tendency to overfit compared to more shallow approaches – meaning that the model does not generalize well for new datasets (as opposed to training data) (39). In other fields of applications, DNNs often undergo extensive validation on new (aka external) datasets to evaluate their real performance – however, in mental health, especially in psychosis research, such validations are missing due to the scarcity of data (39). Therefore, the evaluation of the performance of such models in the current literature is challenging.

### 4.1 Measuring semantic coherence and semantic density based on latent semantic analysis or word-embedding

This approach involves analyzing the semantic coherence and meaning by examining word associations and relationships. This approach is built on Latent Semantic Analysis (LSA) or on word-embedding. LSA is a computational technique used to analyze and represent the meaning of words and documents based on their patterns of co-occurrence in a large corpus of text. It is a statistical method that aims to capture the underlying semantic structure of language by identifying latent (hidden) relationships between words and documents. LSA operates on the principle that words that appear in similar contexts are likely to have similar meanings, whereas words that do not appear in similar contexts are likely to have different meanings. LSA involves creating a matrix that represents the co-occurrence frequencies of words in a text corpus (40). This matrix is then transformed using a mathematical technique called Singular Value Decomposition (SVD) to reduce the dimensionality of the data and extract the most important latent semantic dimensions. These dimensions represent the underlying themes or topics in the text corpus. By projecting words and documents onto these dimensions, LSA can measure the similarity between them based on their semantic content.

On the other hand, word embedding is a more recent approach that also learns distributed representations of words based on their contextual usage. It is typically achieved through neural network models such as Word2Vec, GloVe, or BERT. Word embedding algorithms consider the local context of a word within a sentence or text window and aim to encode its meaning as a ‘dense vector’ in a high-dimensional space. In this lexicon, a dense vector serves as a specialized encoding mechanism for signifying the semantic essence of a given word within a computationally amenable framework. It posits a representation akin to assigning a distinctive coordinate to each word within a high-dimensional space, where the spatial arrangement encapsulates the nuanced semantics of the word. The term “dense” underscores the information-rich nature of these vectors, encapsulating multifaceted information of the word’s semantic domain. In operational terms, word embedding algorithms scrutinize the localized context of a word within a sentence or a proximate textual window. The objective is to transmute the word’s semantic import into a dense vector residing within a multi-dimensional space. Through iterative training processes, these vectors undergo adjustments to position akin words in closer proximity within the vector space, thereby effectively delineating semantic interrelations among words (similar words closer to each other in the vector space). Therefore, similar to LSA, word embedding creates a metaphorical ‘map’ of words in relation to each other, on which the actual text that researchers are interested in can be projected.

LSA and word-embedding have been applied in various areas of Natural Language Processing. In the context of psychosis, they have been used to assess semantic coherence. By comparing the semantic similarity between words or sentences researchers can quantify the extent to which language exhibits disorganization or lack of coherence. For example, Elvegag and colleagues found that LSA derived coherence scores were sensitive to differences between psychosis patients and controls, and that these coherence scores correlated with clinical measures of thought disorder (41). LSA could also be used to localize where incoherence occurs in a sentence, predict levels of incoherence. Measures derives from LSA could be used to predict whether a given discourse “belonged” to a patient or control (9). In another study, LSA measures strongly correlated with clinical rated symptoms of derailment and tangentiality as coherence measures and number of words were both negatively correlated to these symptoms (42). Furthermore, (43) found that semantic coherence, and another LSA-based measure of whether participants response deviates from a ground-role description (called “On Topic” in the manuscript) differed between subjects at clinical high risk for psychosis, first episode psychosis and healthy controls (43). Studies that applied semantic features could also predict transition to psychosis in people who were at risk of psychotic disorders with 80–90% accuracy (15, 31, 32).

### 4.2 Measuring syntactic complexity and syntactic changes based on part of speech tagging

Part of speech (POS) tagging is a linguistic technique that assigns grammatical categories to each word in a sentence. The purpose of POS tagging is to identify and categorize words into their respective parts of speech, such as nouns, verbs, adjectives, adverbs, pronouns, prepositions, conjunctions, and interjections.

The process of POS tagging involves analyzing the linguistic context of each word in a sentence and determining its appropriate part-of-speech tag. This is typically done by using pre-trained statistical models or machine learning algorithms that have been trained on annotated corpora.

Part of speech tagging can be performed using rule-based approaches, where specific grammatical rules and patterns are used to assign tags based on the word’s context. However, more commonly, statistical models, such as Hidden Markov Models (HMMs) or Conditional Random Fields (CRFs), are used. These models learn from labeled training data where each word is associated with the correct POS tag, and then they use this knowledge to predict the tags for unseen words.

The output of a POS tagger is a sequence of tags, with each tag corresponding to a word in the input sentence. For example, given the sentence “The cat is sleeping,” a part-of-speech tagger might produce the following tags: “DT NN VBZ VBG.” Here, “DT” stands for determiner, “NN” stands for noun, “VBZ” stands for verb (third person singular present tense), and “VBG” stands for verb (gerund or present participle).

By analyzing the syntactic structure and changes in sentence patterns using POS tagging, researchers can quantify syntactic complexity and identify deviations from normal speech patterns. Syntactic complexity refers to the level of intricacy and sophistication in sentence construction, such as the use of complex sentence structures and the arrangement of words and phrases. Deviations in syntactic patterns may indicate disruptions in language processing and production. Stanislawski et al. examined the association between negative symptoms and syntactic features and found that determiner pronoun use was significantly negatively correlated with negative symptoms (44). Syntactic measures were also correlated with negative symptoms (Positive and Negative Syndrome Scale) and cognition (Brief Assessment of Cognition in Schizophrenia) in a Dutch-speaking sample (45, 46). In an Indonesian sample, syntactic features showed changes in clinical high-risk subjects compared to healthy controls (47). In longitudional settings, syntactic complexity deteriorated within the 6 months following the first episode of psychosis in those who developed a diagnosis of schizophrenia (48). Moreover, in a clinical high-risk cohort, syntactic features, combined with semantic coherence could predict psychosis onset and frequency of types of “complementizer” words such as “that” and “which” were negatively correlated with negative symptom severity (31).

### 4.3 Measuring speech connectivity based on graph theory

Graph theory provides a framework for analyzing the connectivity and relationships between linguistic elements in speech. By representing speech as a network of interconnected nodes (words or phrases) and edges (relationships between them), researchers can examine the flow of information, identify key nodes, and detect disruptions in the connectivity patterns within the speech. Mota et al. first used graph-based analysis to study speech connectivity and connect it to formal thought disorder (49). Different features of speech connectivity have been found to distinguish schizophrenia patients from patients with mania with up to 93.8% of sensitivity and 93.7% of specificity (50). These connectedness features were found to be informative about of negative symptoms score, predict symptom severity and schizophrenia diagnosis with 91.67% accuracy and 85% accuracy 6 months in advance (30). Novel methods have recently been developed combining connectivity and semantic approaches using semantic networks which showed significant differences between first episode patients, healthy control and clinical high-risk groups (51).

### 4.4 Extracting different acoustic features from audio files

Signal processing techniques are used to extract various acoustic features from audio recordings. These features include temporal (e.g., speech rate, pauses, rythms), spectral (e.g., frequency distribution), loudness (e.g., intensity), phonation (e.g., voice quality, shimmering) and frequency (e.g., pitch) characteristics. Temporal features capture the timing and rhythm of speech, spectral features provide information about the frequency content of speech, loudness features relate to the intensity or volume of speech, and frequency features refer to the pitch or tonal characteristics of speech. Software tools like OpenSmile from Audeering and Praat are commonly used for extracting these features, which provide insights into the acoustic properties of speech. Authors often perform feature engineering to extract low-level descriptor feature sets, either specifically designed for the study [e.g., (52)] or use feature sets that has been already established and tested in the research community. From the latter one, a feature set, called eGeMAPS (Geneva Minimalistic Acoustic Parameter Set for Voice Research and Affective Computing) is especially popular as it has been designed to capture emotional information from speech by the combined effort of speech researchers (53). For example, it reached an accuracy of 82.8% in classifying patients with schizophrenia and healthy controls in a Dutch study (35). In the same study, positive, negative and general psychotic symptoms scores were correlated with acoustic features like pitch, formant frequencies and length of voiced and unvoiced regions. Acoustic measures could accurately capture negative symptoms like blunted vocal affect and alogia in another study (54). Acoustic parameters could also capture negative prodromal symptoms (measured on Structured Interview for Prodromal Syndromes/Scale of Prodromal Symptoms) (55) and identify individuals who transition to psychosis from clinical high-risk state with high accuracy. Importantly, these acoustic parameters, unlike former measures, have been tested in settings when they had to discriminate between multiple types of conditions above healthy control and psychosis (like major depression, anxiety, personality disorder) and demonstrated acceptable discriminatory power (52, 56, 57). As psychosis often occurs together with comorbid conditions, it is important to explore whether speech abnormalities are uniquely associated with psychotic symptoms and these findings suggest the existence of these specific features, supporting the translational potential of speech-based assessment to clinical practice.

### 4.5 Deep neural networks applied on audio data or spectrograms

Deep neural networks (DNNs) are a type of artificial neural network (ANN) that are designed to model and learn complex patterns and relationships within data. They are inspired by the structure and functioning of the human brain, specifically the interconnected network of neurons. In a DNN, information is processed through multiple layers of interconnected nodes, known as neurons. These layers are organized hierarchically, with each layer extracting and transforming features from the input data. The initial layers learn low-level features while subsequent layers learn higher-level features. The final layer provides the output or prediction based on the learned features.

The term “deep” in deep neural networks refers to the presence of many “hidden” layers in the network architecture (layers between input and output). Unlike shallow neural networks with only one or two hidden layers, deep neural networks can have tens, hundreds, or even thousands of hidden layers. The depth of the network allows for the representation of increasingly abstract and complex features, enabling the network to capture intricate patterns in the data and learn non-linear decision boundaries. DNNs are trained through a process called backpropagation, where the network adjusts the weights and biases of its connections to minimize the difference between its predictions and the desired output. This training is typically performed using large, labeled datasets, allowing the network to learn and generalize from the examples provided.

In psychosis research, DNN-s have been only applied in the vocal domain and to the authors knowledge, there are currently only four studies available. Amiriparian et al. (58) focused on audio-based recognition of relapse state (mild, moderate, severe) of bipolar disorder using capsule networks, a type of neural network architecture designed to capture hierarchical relationships between features. The researchers first created spectograms from audio signals and then extracted features of them. Garoufis et al. (59, 60) utilized unsupervised learning with Convolutional Variational Autoencoders (CVAE), a type of generative model, to learn latent representations of speech data. By comparing the reconstructed speech to the original, the model identified deviations that may indicate relapse episodes. Their studies demonstrated the feasibility of unsupervised learning methods for detecting relapses based on speech characteristics, without requiring labeled data or subject-specific models – although it is important to note that these findings based on preliminary data from an ongoing study, therefore have been tested with limited sample sizes (*N* = 5 and *N* = 13) (59, 60). Fu et al. (36) focused on schizophrenia and proposed an end-to-end architecture, called Sch-net for automatic detection of schizophrenia from speech. Sch-net - similarly to Amiriparian’s approach (58) – utilizes a convolutional backbone architecture applied on spectrograms but adds two specific components, skip connections and convolutional block attention module (CBAM) to it. The skip connections are designed to enrich the information used for the classification by emerging low- and high-level features while the CBAM highlights the effective features, therefore avoiding the procedure of manual feature extraction and selection. Their end-to-end solution reached excellent accuracy (98%) when discriminating between healthy controls and schizophrenia patients.

## 5 Speech techniques and analysis applied in subclinical populations

Studies by Bedi et al. (31) and Corcoran et al. (32) demonstrated that individuals at clinical high risk state exhibit alterations in language use compared to healthy controls. Their language features have been shown to identify individuals who will eventually develop clinical psychotic disorders from ones who remain in subclinical stage with high accuracy. These studies found that individuals with subclinical psychotic symptoms exhibited reduced semantic coherence and increased syntactic errors in their speech, suggesting disruptions in higher-order language processing. People at clinical high risk also showed lower level of speech connectivity compared to healthy controls when speech graphs has been used to analyze their speech, especially if they developed clinical psychosis later (11). Also, within the context of subclinical psychosis, studies using schizotypy as a framework have provided valuable insights [to the deeper understanding of schizotypy concept, the authors suggest reading the work of (61–63)]. High level of schizotypy has been associated with alterations in speech production, speech variability, and speech content (64–66). Furthermore, studies have demonstrated that individuals with high schizotypy exhibit alterations in acoustic features of speech like reduced speech variability, expressiveness and atypical pitch patterns compared to those with lower schizotypy scores (20, 67). In remotely collected speech samples, schizotypy scores were positively associated with acoustic features like Mel frequency cepstral coefficients (MFCC), loudness parameters, Hammerberg –index, Spectral flux, and slope measures (68–70), where changes in loudness parameters were uniquely associated with schizotypy (compared to features associated with anxiety and depression symptoms). Importantly, analytical and modelling approaches that have been used to discriminate clinical psychosis from healthy controls could be successfully applied to discriminate between low and high level of schizotypy, reaching 69–88% of accuracy (68–70). These findings suggest that speech analysis techniques hold promise in capturing subtle speech abnormalities associated with subclinical psychotic symptoms.

## 6 Current research challenges

### 6.1 Sample size and sample bias

A key challenge in speech and psychosis research lies in samples. Previous work was conducted in small samples, i.e., less than 50 participants per group [e.g., (15, 30–32, 36, 55)]. Generalizability appears to be an outstanding concern as many articles in the fields applied machine learning methods that are prone to overfitting. As former samples were often non-balanced, including only a handful number of participants in categorical groups (e.g., (30, 31)) these findings require large-scale, more generalizable replications on external datasets.

Furthermore, sample size not only raise concerns in terms of generalizability, but they also limit scientific exploration. Specifically, studies generally do not have sufficient statistical power to compare groups alongside the high number of features that can be extracted from speech which makes it difficult to assess differences between speech pattern in their full complexity. For example, most studies [e.g., (11, 30–32, 52, 54, 57)] apply more than 10 features. Even calculating with 10 features, medium effect sizes, a statistically powered analysis of comparing means of these features between two groups would require a sample size of 468 participants (234 per group). If researchers wanted to evaluate vocal differences alongside the popular eGEMAPS feature set (see details in Section 4/Extracting different acoustic features from audio files), a statistically powered comparison between two groups would require a sample size of 726 (353 participants per group). Clinical studies do not operate with such sample sizes, given feasibility constraints.

Another form of limitation is the limited types of machine learning algorithms that can be applied on the samples as some models, especially DNNs cannot reach their full predictive potential without sufficient amount of training data. Therefore, the limit in sample sizes is also the limit of exploring how much information can be captured from speech in relation to psychosis.

Independent from sample sizes, other problems that stem from research samples are limited representability and generalizability. Research samples and therefore training data in machine learning model are often unrepresented for patient population in terms of demographic information like ethnicity, gender, education level or location. Machine learning models tend to make more accurate predictions for subgroups that have more examples in the training corpus which might lead to bias in less represented groups. However, studies do not report model performance specified in different subgroups that leaves the field with no information about this effect. Another challenge around representability is that most of these studies carried out dichotomous comparisons between small samples of completely healthy subjects and stereotypical patients, in whom the effects might be most apparent, but findings are not applicable for real-life conditions when people are presented with a wide range of symptom severity.

Conducting research in subclinical populations, especially in the general population is a feasible solution to overcome these problems. Although alongside with more subtle symptomology we can assume more subtle alterations in speech and hence a larger required sample size because of smaller effect sizes, the frequency of the investigated phenomena is much higher and the barrier to get access to these populations is much lower (e.g., easier to collect their speech in online, remote settings; easier to recruit) that can lead to bigger and more representative samples. Another solution can be collecting short speech samples in standardized, prompt based-settings [several feasible methods have been proposed by (43, 50, 51)] or by simply recording clinical interviews. These solutions combined with the application of automated transcription of voice into text (45, 46, 68–70) can reduce the cost of, and accelerate the speed of data collection. Complementary to subclinical research, data sharing and publication of datasets is another community-level effort that should be taken to overcome research limitations stemming from samples (Table 3).

**Table 3:** Summary of current challenges in speech and psychosis research and how subclinical studies can help to tackle them.

**Challenges**	**How subclinical research can help to overcome it**
**Sample size and sample bias**	Conducting research in subclinical populations, especially in the general population can increase sample sizes and involve unrepresented groups
**Lack of longitudinal observations**	Subclinical studies can enable the observational of the natural progression of the illness from early stages, identify protective, triggering and risk factor; reduce cost and facilitate more frequent follow-ups
**Lack of standardization and transparency**	Subclinical research can experiment with different methodologies to establish standardized procedures for speech elicitation and analysis. Once established, these procedures can be applied to clinical samples.
**Cross-language and cross-cultural barriers**	Studying subclinical samples can help develop standardized assessments and pipelines for cross-cultural and cross-language research.
**Co-morbidities and transdiagnostic perspective**	Subclinical research allows for the examination of speech abnormalities across different symptomologies. Studying subclinical samples might also help unraveling the complex interactions between speech disturbances, co-morbidities, and functional outcomes.

### 6.2 Lack of longitudinal observations

Longitudinal studies play a crucial role in understanding the dynamic nature of psychosis and its associated speech abnormalities. However, the field of speech and psychosis research has been limited by a lack of longitudinal observations. For example, except from studies aim to predict transition or relapse [e.g., (31, 32)], researchers utilized cross-sectional comparisons. Even in those cases, studies applied assessments of only two or three time points instead of continuous, systematic follow-ups. It is crucial to overcome this as longitudinal, continuous studies allow for the examination of changes in speech patterns over time, providing a comprehensive and currently lacking understanding of the evolution and stability of speech abnormalities in individuals with psychosis. These observations, especially if started in the early, subclinical stage could facilitate the identification of early markers and predictive patterns in speech that may differentiate individuals at risk of developing psychosis from those with established psychotic disorders (31). Longitudinal assessments of speech after diagnosis can aid in monitoring treatment response, predicting relapse, and assessing the effectiveness of interventions targeted at improving speech and communication deficits in psychosis (1, 28). Furthermore, longitudinal assessment and analysis techniques are crucial to enable personalized prediction and evaluation instead of current, group-based approaches.

Conducting longitudinal studies in the context of clinical psychosis research can be challenging due to factors such as participant attrition, lengthy follow-up periods, and drop-out (6). Also, longitudinal studies often require significant resources, including funding, personnel, and infrastructure, which may pose obstacles to their implementation (71).

However, like challenges around sample sizes and biases, encouraging collaboration among research institutions and establishing data sharing initiatives can help overcome the limitations of individual studies and facilitate the accumulation of longitudinal speech data in psychosis research (71). Leveraging advancements in technology, such as smartphone applications or online speech collection, can enable remote and continuous monitoring of speech patterns, enhancing the feasibility and scalability of longitudinal studies in this field (72, 73). Subclinical studies are particularly suitable for longitudinal assessment, as it can enable the observation of the natural progression of the illness from early stages, identify protective, triggering and risk factors and can reduce costs as certain participants may not need clinical interventions. Online and remote, time-efficient assessment of symptoms and speech can increase feasibility more, leading to larger samples at a reduced cost, and possibly enabling more frequent follow-ups (Table 3).

### 6.3 Lack of standardization and transparency

Another challenge is the lack of standardization and transparency in assessment, endpoints, methodologies and analysis techniques which hinder the comparability and reproducibility of findings across studies.

Firstly, there is a lack of standardized protocols for data collection and speech assessment in psychosis research. Different studies use diverse speech tasks, prompting participants to engage in varied conversational, narrative scenarios or simply record interviews or phone calls with clinicians. The choice of prompts and tasks can significantly impact the content and quality of speech produced by individuals with psychosis (43, 50, 72, 73). This variability makes it difficult to compare and combine results across studies as well as conduct external validation of models (71). By using consistent prompts across studies, researchers could ensure that participants are engaging in similar speech scenarios, allowing for more meaningful comparisons of speech features.

In addition to this, researchers tend to extract different feature sets or develop new features in much of their work. Whilst this may increase knowledge and expand methodological choices, it also limits comparability, especially in combination with different speech elicitation procedures. For instance, one study might focus on semantic coherence during a picture description task, while another might examine syntactic complexity in a spontaneous speech task. In addition to this, studies often fail to combine different types of features, but focus on one aspect of speech – for example extracting semantic and syntactic changes without analyzing vocal parameters. Given the complexity of speech as a signal and the complementary nature of features predictive models could be improved by more comprehensive and multi-layer assessment of speech (43, 71, 74). Neglecting the involvement of a wide range of features not only prevents meaningful comparison of findings but limits the exploration of the full-potential of speech-based assessment.

Furthermore, there is a need for greater transparency in reporting the details of speech analysis techniques and algorithms used in research. Many studies fail to provide a thorough description of the algorithms employed for analysis or the validation procedures. This is especially problematic in the case of deep learning models when fine-tuning and hyperparameter optimalization plays a crucial role. Without clear documentation, it becomes challenging to replicate, compare, evaluate or externally validate the findings. To increase replicability, it would be crucial to provide detailed information on the preprocessing steps, feature extraction methods, and machine learning algorithms used and provide a well-documented, public code base.

Research conducted in subclinical samples could be an ideal and cost-effective way of experimenting with different methodologies in order to establish a widely accepted, scalable procedure for speech elicitation and analysis which later, can be applied on clinical samples (Table 3).

### 6.4 Cross-language and cross-cultural barriers

Cross-language and cross-cultural barriers present other significant and unsolved challenges. Language and cultural factors can influence speech patterns, speech features, communication styles, and the interpretation of speech abnormalities, making it crucial to consider these aspects in research (75–78). One challenge in cross-language and cross-cultural research is the availability of standardized assessments and linguistic resources in different languages. Many speech analysis tools and measures have been developed and validated predominantly in American and British English, limiting their applicability to other linguistic contexts. Studying speech-abnormalities and different methods across languages and cultures can provide insight into main, robust and disorder specific speech changes. As cultural norms and communication styles can vary across different cultures, impacting the expression and perception of speech abnormalities – therefore applying standard recruitment, inclusion and exclusion criteria, assessment and analysis pipeline would be essential in cross-cultural and cross-languages context. Applying such pipelines is much more feasible in subclinical samples not only because of the wider – range of available sample population but also because by recruiting from general population researchers can avoid the national, cultural, financial and regulatory differences between psychiatric clinics and care that could bias the sample and delay research procedures (Table 3).

### 6.5 Co-morbidities and transdiagnostic perspective

Psychosis often co-occurs with other mental health conditions, such as mood disorders, anxiety disorders, and substance use disorders. This co-occurrence poses challenges in understanding the unique contributions of speech abnormalities to psychosis and its specific disorders. However, this challenge is often overlooked by studies that only compare healthy control groups with psychotic disorder groups. It is highly problematic for several reasons. Firstly, this unnaturalistic setting that does not mimic the day-to-day challenges of real-world clinical assessment. Real-world patients present complex symptomology and clinicians barely struggle with discriminating between healthy and psychotic individuals but rather with assigning correct differential diagnosis/diagnoses and assessing the severity of symptoms and risks or judging potential treatment response. Secondly, findings from such study designs cannot provide sufficient information to decide whether the given methodology was able to discriminate unique features of psychosis from speech or rather just captured a broader difference between healthy and not healthy speech patterns. Thirdly, it limits researchers to identify and distinguish disorder-specific and transdiagnostic changes in speech.

Research on subclinical samples allows for the examination of speech abnormalities across sub-clinical-level symptoms and different diagnostic categories. This approach might identify common speech markers that may cut across various mental health conditions. For example, features like reduced number of words, reduced duration, spectral changes, lower pitch, decrease in clear articulation and speech connectivity have been observed in other psychiatric conditions like major depression, anxiety, PTSD, cannabis use or ADHD (75, 79, 80). If these speech characteristics also occur in subclinical samples, that might signal shared psychopathology between these (often co-morbid) conditions and subclinical psychotic symptoms and at-risk mental state of psychosis /ultra-high risk of psychosis that are reflected in speech patterns. Therefore, identifying similar patterns in speech alteration can help researchers to form hypotheses about how subclicinal psychotic symptoms relate to other mental disorders.

Furthermore, studying subclinical samples helps unravel the complex interactions between speech disturbances, co-morbidities, and functional outcomes. Individuals with subclinical psychosis-related speech abnormalities may exhibit different patterns of co-morbidities compared to those with clinical psychosis. Exploring the relationship between speech abnormalities, co-morbidities, and functional outcomes in subclinical populations can provide insights into the dynamic relationship of speech disturbances with the developmental course of different mental health conditions in an ecologically valid way and at low costs (Table 3).

## 7 Conclusion

Automated speech analysis techniques can capture and aid in the prediction of a wide range of psychosis symptomology, including subclinical symptoms. These techniques also hold promise in the longitudinal prediction of transition to clinical psychosis from at-risk state, and relapse or treatment response in people with clinical level psychosis. Despite the high potential and the wide range of possible clinical applications, translation into practice is hampered by numerous challenges in scientific validation. These include small, unrepresentative research samples, unstandardized assessment and evaluation protocols, lack of reproducibility, transparency, data sharing, external validation, lack of cross-cultural and cross-language explorations, transdiagnostic exploration and cross-diagnostic comparison and absence of longitudinal, continuous studies. Introducing more research on automated speech analysis techniques in the subclinical population can help to overcome these challenges by decreasing research costs, increasing access to more representative and diverse samples, increasing feasibility and enabling a developmental insight into the emergence of speech abnormalities. Eventually, sub-clinical research can also serve as a way to test hypothesis and methodologies, that subsequently, clinical research can specifically focus on.

## Author contributions

JO: Conceptualization, Investigation, Project administration, Visualization, Writing – original draft, Writing – review & editing. TS: Conceptualization, Methodology, Supervision, Writing – review & editing. NC: Supervision, Writing – review & editing. KD: Conceptualization, Funding acquisition, Investigation, Supervision, Writing – review & editing.
